# Clinical Reports of the Demilt Dispensary

**Published:** 1878-07-20

**Authors:** L. Duncan Bulkley


### CLINICAL REPORTS OF THE DEMILT DISPENSARY.

BY DR. L. DUNCAN BULKLEY.

*Seven cases of Palmar Syphilis.*—When affecting the palms of the hands and the soles of the feet alone, syphilis is so commonly mistaken for other diseases that a brief mention of seven cases which have been under treatment during the past year may not be without interest and profit. It is well known that palmar eczema and psoriasis may simulate the eruption caused by syphilis so perfectly that it is often a matter of great difficulty to make the diagnosis from the appearance of the eruption alone. Several of the cases here reported had long passed unrecognized, one of them having been twice in a hospital, for periods of three and six months respectively, without the true nature of the affection having been recognized, the treatment being purely local, and no investigation of the constitutional nature of the disease being made; and moreover, the effects of all previous treatment had been but moderate and transitory.

The lesions of syphilis may appear upon the palms and soles either early after infection, or as one of the very late manifestations of the poison (I have observed it there as late as twenty years, as far as could be made out, after the disease has been acquired), and the cases present somewhat different appearances, according as the eruption is one of the early or late symptoms of the disease. In the earlier cases the lesions are apt to be multiple, as all the earlier manifestations of syphilis, whereas eruptions on the palm or sole late in the history of syphilis partake more of the nature of the latter lesions, and are more commonly single. These seven cases may be thus divided, and I will mention first the three in whom the palmar and plantar exhibitions of syphilis occurred early in the disease.

Case I.—Joseph Kelly, aged thirty-three, came to Demilt, February 27, 1877, saying that he had had a chancre on the penis four months previously, which was followed, at the expiration of about a month, by a general, scattered, papular eruption. Examination revealed the remains of a chancre at the end of the meatus; there was somewhat a depression and some hardening still remaining, with moderate inguinal adenopathy.

The centres of both palms were the seats of a distinctly tubercular and squamous eruption, with well-defined margins passing abruptly from diseased to healthy tissue; the patches were about an inch and a half in diameter, and nearly circular. On the feet there was a similar eruption of flattened, scaly tubercles, disposed in a more or less circular form, about in the centre of each sole. The color of the eruptions was of a dark red where the outer layers of epidermis were gone, but, where this still was intact, they had a dirty-yellow look, with punched-out edges. There had never been any moisture, and little if any itching. There was still some of the general eruption to be seen on the legs, and some superficial redness of the fauces existed.

He was put under the use of mercurial inunctions into the sides and thighs, with no local treatment, and on March 1st it was noted that there had been very great improvement in the eruption everywhere. He continued to improve, but after some weeks became careless, and returned on April 14th, after an absence of three weeks, with the eruption worse. On May 5th a sclero-choroiditis was discovered.

He continued under treatment some weeks longer, and the eruption nearly disappeared, when he again failed to attend, and has not been seen since.

Case II.—A woman, J. H., aged forty-three, acknowledges to having had sores in the vulva in March, 1875, which were soon followed by a general eruption, the palms being affected at the same, or about the same, time. She has been in attendance at the Dispensary nearly a year; she has a family, and as soon as the hands improve to a certain point she always neglects treatment, and returns when they give her annoyance.

In her case the eruption occupied the palms of both hands, being made up of isolated tubercles, somewhat elevated, fissured here and there with a small amount of scaling. The edges of the eruption, which were made up of separate papulo-tubercles, in some places touching each other, were sharply defined, as if punched out, and, on raising the epidermis, which was loosened from the inner border, it was seen to run externally down into healthy tissue beyond the eruption.

She was given a mixed treatment of bichloride of mercury and iodide of potash, with no local measures, and the improvement was very prompt and decided, but, like so many of these patients, attendance became irregular as the disease gave less trouble, and at the last note, May 24th, there were still some traces of the lesion, in the way of a few small fissures where former tubercles had been.

This woman has five healthy children, born before the occurrence of the chancre.

Case III.—This patient gives no history of infection, but the appearance of the eruption on the palms is that of a more recent syphiloderm, and the following history of her pregnancies confirms this. She has been married twice and had twelve children by her first husband, and none by the second; all the children came to full term except one which miscarried at three months, the cause of which she attributed to fright. All the children are healthy but one, who is under treatment for eczema of the leg.

When first seen both palms were the seat of eruptions corresponding mainly in appearance to those described in the two preceeding cases. On the right palm there was a clearly-defined, irregular patch about one by one and a half inch in diameter, seated at the roots of the two middle fingers. On the palmar surface of the left hand there were three distinct patches at the roots of the last three fingers. She was given inunctions of mercurial ointment into the sides and thighs, and improvement resulted, but she also was careless, and the case was not followed to the end.

The following cases are instances of the eruption of syphilis occurring on the palm and sole later in the disease.

Case IV.—Ann McC., aged twenty-nine years, has every appearance of health, with the exception of the single lesion on the right palm, which, when she first came for treatment (October, 1876), almost incapacitated her for work; the disease occupied the larger portion of the internal surface of the hand, including the fingers and thumb. Most of this surface was bereft of epidermis, was of a deep, purplish red, the larger part of it smooth and shiny, with here and there separate or grouped tubercles, on which the new epidermis was beginning again to harden and peel; where these tubercles existed at or near flexures, there were cracks, which were very painful. The margins of the eruption were very clearly defined, punched out in appearance, with the edges of the epidermis everted, and when this was pulled on toward the healthy tissue it could not be separated without giving pain. On closer examination the greater part of the margin could be recognized as composed of separate tubercles arranged side by side, in some instances running into each other, at others quite isolated.

Her history was perfectly conclusive of the nature of the disease: her first husband confessed to having syphilis, and ten years ago she became infected and had the usual phenomena, obstinate sore throat, loss of hair, occipital headache in the afternoon, and three miscarriages at two, three, and five months respectively, followed by a dead child at full term, and two other children which lived but a few weeks, and had eruptions which, from the description, were syphilitic. The syphilitic lesion on the palm did not appear until three years before her visit to me, or *seven years* after she acquired the disease.

She was placed upon a mixed antisyphilitic treatment, and made very great improvement without any local measures; subsequently, however, these were added to expedite the cure and to counteract the effect of her occupation. She also was rather fitful in attendance as the hand approached a cure; and at the last note, June 14th, there were still some remains of isolated tubercles, although the hand has long ceased to give her any great annoyance. She was obliged to do [illegible] work and washing most of the [illegible]e under treatment, which delayed the recovery greatly. I may mention, in this connection, that another patient treated some time ago, who was a silver-plater, and obliged to keep the hands in acid much of the time, recovered completely and remained well of a palmar syphiloderm, he taking only the mixed treatment internally, with no local measures whatever.

CASE V.—Mary D., aged sixty, a widow woman, has always enjoyed good health until a year ago, when, as she states, after smoking the pipe of a boarder, she acquired a general papular eruption, which was preceded by a sore throat, and followed by sore eyes and falling of hair. When first seen, June 9, 1877, the eruption was confined chiefly to the left hand, the right being entirely free. The lesion occupied an elliptical surface, extending from a little below the wrist, over the ball of the thumb, around to the roots of the fingers and along the ulnar border of the hand, joining the starting point just above the wrist; the centre of the palm appeared free. The eruption was made up of tubercles, slightly raised above the level of the skin, which in some places touched and coalesced; there was considerable loss of the epidermal layer on the affected portions, with sharply-cut edges.

On the radial side of the left arm near the elbow, also on the right forearm, and on the back of the left hand, were the remains of a papulo-tubercular eruption, of dark red color, and on the anterior surface of the right leg were brownish stains of a similar lesion. She was placed upon a mixed treatment with immediate improvement to the eruption, no local measures being employed.

CASE VI.—James McK., aged fifty years, gives no history of contagion, the first lesion of syphilis which he acknowledges to being the present eruption, which he says came first a year and a half ago; the palmar lesion appeared to be but a portion of the papulo-tubercular eruption existing elsewhere. On the surface of the right foot, extending around on to the tendo Achillis, also on both legs, and to moderate extent on the arms, there was an eruption disposed to a great extent in the form of circles and gyrations, composed of flat papulo-tubercles, in many instances touching each other, in others isolated, of a deep, coppery-red color, and with but moderate scaling. On the right palm was an eruption composed of the same elements, and exhibiting characteristics corresponding to those previously detailed, which need not be repeated, as the lesion presented nothing unusual. He was seen but once.

CASE VII.—James Higgins, aged forty-two, a laborer, appeared at the Dispensary first on January 27, 1877, for the treatment of an affection of the sole of the foot, which had lasted a year and a half. He is a man of more than ordinary intelligence for his class and occupation, and denied having had syphilis, and was much surprised when the disease was confidently ascribed to this cause; he gave no history of syphilis as far as could be made out.

On the middle of the sole of the right foot there existed an irregularly-shaped patch, with circular edges, of diseased tissue, from which most of the normal epidermal covering was gone. On close examination it was seen to be composed mainly of separate elements, irregularly placed, which presented themselves as papular or small tubercular prominences, with a dirty-grayish epidermal covering. The margins of the patch were very clearly defined and sharply cut, the epidermis standing up at the edge, and when pulled on the layer was found to reach down into healthy tissue peripherally.

For the purpose of clinical study, the case being carefully watched by a number of medical gentlemen attending the clinic, he was given only mercurial ointment, for inunction into the sides and thighs, with no local application whatever. The patch fairly melted away under the anti-syphilitic treatment; in one week, on February 3d, it was discovered that there was great improvement; on March 3d it was noted that the eruption was nearly gone; and on March

13th, or six weeks from the first visit, the record reads that there was only staining left; the patch had existed one and a half year previously.—*N. Y. Med. Journal.*

